# Early recognition and treatment of neuropsychiatric symptoms to improve quality of life in early Alzheimer’s disease: protocol of the BEAT-IT study

**DOI:** 10.1186/s13195-019-0503-2

**Published:** 2019-05-24

**Authors:** Willem S. Eikelboom, Ellen Singleton, Esther van den Berg, Michiel Coesmans, Francesco Mattace Raso, Rozemarijn L. van Bruchem, Jeannette A. Goudzwaard, Frank Jan de Jong, Marc Koopmanschap, Tom den Heijer, Jan J. M. Driesen, Lilian J. H. M. Vroegindeweij, Elsbeth C. Thomeer, Susanne E. Hoogers, Anke A. Dijkstra, Sytse U. Zuidema, Yolande A. L. Pijnenburg, Philip Scheltens, John C. van Swieten, Rik Ossenkoppele, Janne M. Papma

**Affiliations:** 1 Department of Neurology, Erasmus MC University Medical Center, PO Box 2040, 3000 CA Rotterdam, the Netherlands; 2 Department of Neurology, Alzheimer Center Amsterdam, Amsterdam University Medical Center, PO Box 7057, 1007 MB Amsterdam, the Netherlands; 3 Department of Psychiatry, Erasmus MC University Medical Center, PO Box 2040, 3000 CA Rotterdam, the Netherlands; 4 Department of Internal Medicine, Erasmus MC University Medical Center, PO Box 2040, 3000 CA Rotterdam, the Netherlands; 5 Erasmus School of Health Policy & Management, Erasmus University, PO Box 1738, 3000 DR Rotterdam, the Netherlands; 6 Department of Neurology, Franciscus Gasthuis, PO Box 10900, 3004 BA Rotterdam, the Netherlands; 7 Department of Neurology, Franciscus Vlietland, PO Box 215, 3100 AE Schiedam, the Netherlands; 8 Department of Neurology, Het Van Weel-Bethesda Ziekenhuis, PO Box 153, 3240 AD Dirksland, the Netherlands; 9 Department of Neurology, Maasstad Hospital, PO Box 9100, 3007 AC Rotterdam, the Netherlands; 10 Department of Neurology, Spijkenisse Medical Center, PO Box 777, 3200 GA Spijkenisse, the Netherlands; 11 Department of Anatomy and Neurosciences, Amsterdam University Medical Center, PO Box 7057, 1007 MB Amsterdam, the Netherlands; 12 Department of General Practice and Elderly Care Medicine, University of Groningen, University Medical Center Groningen, PO Box 30,001, 9700 RB Groningen, the Netherlands; 13 Clinical Memory Research Unit, Lund University, Simrisbanvägen 14, 212 24 Malmö, Sweden

**Keywords:** Alzheimer’s disease, Dementia, Behavior, Caregivers, Neuropsychiatry, Cost-benefit analysis, Prospective studies, Quality of life

## Abstract

**Background:**

Neuropsychiatric symptoms (NPS) are very common in patients with mild cognitive impairment (MCI) and Alzheimer’s disease (AD) dementia and are associated with various disadvantageous clinical outcomes including a negative impact on quality of life, caregiver burden, and accelerated disease progression. Despite growing evidence of the efficacy of (non)pharmacological interventions to reduce these symptoms, NPS remain underrecognized and undertreated in memory clinics. The BEhavioural symptoms in Alzheimer’s disease Towards early Identification and Treatment (BEAT-IT) study is developed to (1) investigate the neurobiological etiology of NPS in AD and (2) study the effectiveness of the Describe, Investigate, Create, Evaluate (DICE) approach to structure and standardize the current care of NPS in AD. By means of the DICE method, we aim to improve the quality of life of AD patients with NPS and their caregivers who visit the memory clinic. This paper describes the protocol for the intervention study that incorporates the latter aim.

**Methods:**

We aim to enroll a total of 150 community-dwelling patients with MCI or AD and their caregivers in two waves. First, we will recruit a control group who will receive care as usual. Next, the second wave of participants will undergo the DICE method. This approach consists of the following steps: (1) describe the context in which NPS occur, (2) investigate the possible causes, (3) create and implement a treatment plan, and (4) evaluate whether these interventions are effective. Primary outcomes are the quality of life of patients and their caregivers. Secondary outcomes include NPS change, caregiver burden, caregivers’ confidence managing NPS, psychotropic medication use, the experiences of patients and caregivers who underwent the DICE method, and the cost-effectiveness of the intervention.

**Conclusions:**

This paper describes the protocol of an intervention study that is part of the BEAT-IT study and aims to improve current recognition and treatment of NPS in AD by structuring and standardizing the detection and treatment of NPS in AD using the DICE approach.

**Trial registration:**

The trial was registered on the Netherlands Trial Registry (NTR7459); registered 6 September 2018.

## Introduction

### Background and rationale

The majority of patients with Alzheimer’s disease (AD) experience neuropsychiatric symptoms (NPS) during the course of their disease [1, 2]. NPS include behaviors such as apathy, agitation, and psychosis, and are already highly prevalent in patients in the early stages of AD including those with mild cognitive impairment (MCI) [3]. NPS have a large impact on the quality of life (QoL) of patients and their caregivers [4], leading to extensive healthcare costs [5]. In addition, NPS are related to accelerated progression of the disease and earlier institutionalization [6, 7].

Although NPS are increasingly recognized as core features of AD [2], NPS are currently underrecognized during the diagnostic phase in memory clinics. This notion arises from our local experience, but one that has also been raised previously by several international research groups [8–12]. While cognitive testing and instrumental activities of daily living (IADL) questionnaires are typically administered during standard clinical work-up, assessment of NPS (e.g., using the Neuropsychiatric Inventory (NPI)) is often not [13]. The failure of clinicians to prioritize the assessment of NPS leads to undertreatment and a variety of associated suboptimal outcomes [14, 15]. This is clearly a missed opportunity since there is growing evidence for the efficacy of psychosocial and pharmacological interventions to reduce NPS and improve QoL in patients with AD [16–19].

NPS are often considered as medication targets in cases where NPS are appropriately detected by clinicians [11]. This leads to high rates of (off-label) psychotropic medication prescriptions that are only modestly effective in dementia [20]. In addition, this symptomatic treatment does not do justice to the multiple contributors causing NPS, including factors relating to the patient (e.g., personality), caregiver (e.g., communication style), and environment (e.g., safety) [11, 21, 22]. Therefore, a patient-centered care (PCC) approach is preferred that considers all these individual factors when managing NPS [1, 17, 23].

After a comprehensive assessment of NPS, nonpharmacological interventions are the first choice to treat NPS in dementia as recommended by the national and international guidelines on the diagnosis and treatment of dementia [24–26]. Although several psychosocial interventions have been developed and proven to be effective (e.g., [27–29]), these programs have rarely been implemented into standard care in memory clinics [10]. Previous studies have suggested various barriers to implementing these guidelines, including a lack of training and knowledge among clinicians regarding the efficacy, dosing, and timing of nonpharmacological interventions [22, 30]. Nonpharmacological strategies are also considered to be more time-consuming compared to psychotropic medication. Furthermore, there are only limited evidence-based interventions suitable for patients with early-stage dementia and their caregivers given the focus of previous research on institutionalized patients with severe dementia [31]. To overcome these barriers, there is a need for a tool that translates the current guidelines into clinical practice and integrates a comprehensive assessment into the standard work-up at memory clinics in order to improve early recognition and tailored treatment of NPS in AD.

Recently, a multidisciplinary expert panel proposed such a tool that integrates current models and theories on the causes of NPS to structure the assessment and management of these symptoms following four steps: Describe, Investigate, Create, Evaluate—i.e., the DICE method [10]. This framework identifies NPS, examines possible underlying causes, and consequently integrates pharmacological and nonpharmacological interventions to treat these symptoms following a PCC approach.

Similar approaches to the DICE method have been developed to address NPS in dementia (e.g., “Grip on Challenging Behaviour” [32], “4D Approach” [33], “Act in Case of Depression” [34], “STA OP!”) [35]. However, studies in community-dwelling patients are lacking, as the majority of these methods have been carried out in the nursing home setting. A recent pilot study showed that the use of the DICE method reduced caregiver distress in caregivers of community-dwelling patients with dementia [36] and supports the use of this approach in the outpatient setting. Moreover, the DICE method has been suggested as the most promising nonpharmacological approach to manage NPS in dementia [37]. Besides the evidence on its effectiveness, demonstrating the cost-effectiveness of the DICE method is crucial before this approach can be part of the standard care [18, 38].

The BEhavioral symptoms in Alzheimer’s disease Towards early Identification and Treatment (BEAT-IT) study is developed to increase our understanding of NPS across the spectrum of AD. This project aims to (1) investigate the etiology of the behavioral variant of AD (bvAD) [39] as a model of the neurobiological mechanisms of NPS in AD and (2) study the effectiveness of the DICE method for the management of NPS in patients with MCI and AD. This paper describes the protocol of an intervention study that focuses on the latter aim.

### Objectives

The aim of this study is to use the DICE method to structure and standardize the recognition of NPS in AD in the memory clinic, implement current guidelines for the treatment of NPS in MCI and AD, and to investigate the effects of the treatment on QoL. Note that we will not evaluate the treatments itself (e.g., the efficacy of psychosocial interventions or antidepressants) since those are already evidence-based interventions recommended by current guidelines, but rather examine the benefits of structuring these interventions in the context of the memory clinic. We will do this by investigating the effectiveness and cost-effectiveness of the DICE method in community-dwelling patients with AD or MCI visiting the memory clinic and compare this group to a control group who will receive care as usual (CAU). We hypothesize that the structuring and standardization of the care of NPS with the use of the DICE approach will improve the QoL of both caregivers and patients at the early stages of AD. In addition, implementing the DICE method is expected to allow early recognition of NPS and reduce NPS, caregiver burden, and psychotropic drug use, and is aimed to be cost-effective. By doing so, this study may contribute to the improvement of early identification and management of NPS in AD in memory clinics.

## Methods

The Standard Protocol Items: Recommendations for Interventions Trials (SPIRIT) guidelines were followed for this protocol [40].

### Study design

**Fig. 1:**
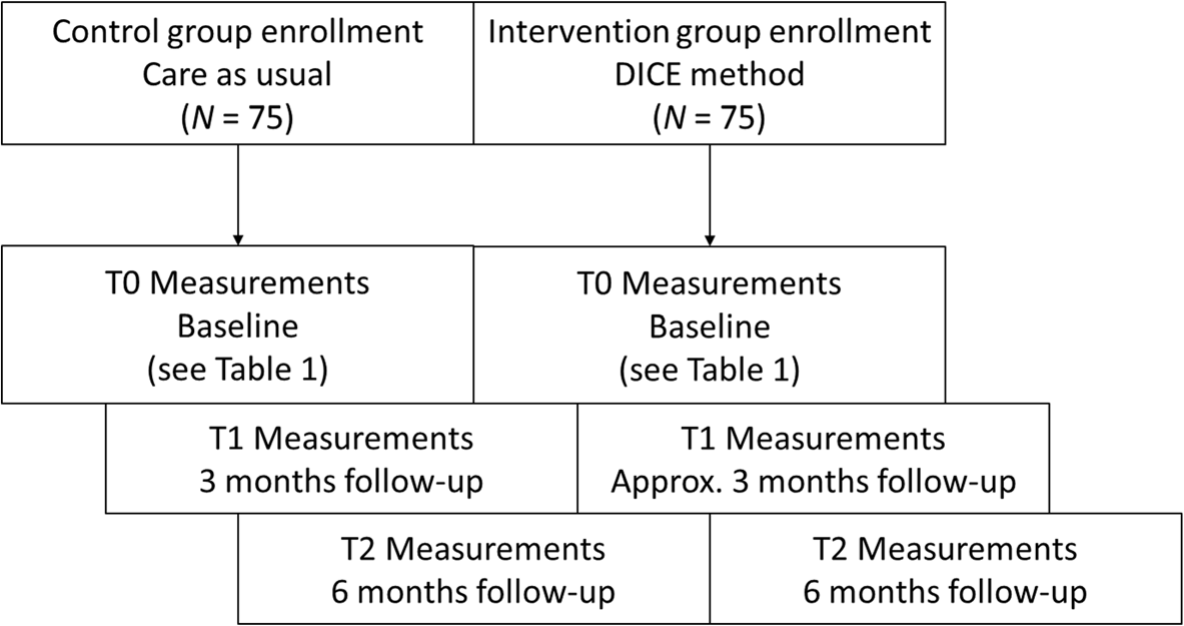
Design of the BEAT-IT study. Note that only the assessments are depicted since intervention visits will vary across subjects in the intervention group due to the personalized approach

This study is a prospective multicenter study with a quasi-experimental design (see Fig. 1). In the first part of the study, a control group will be recruited who will receive CAU. After 1 year, we will enroll the second wave of participants who will receive a structured and standardized assessment and treatment of NPS based on the principles of the DICE method. Hence, the enrollment of the control group will be completed before the start of the inclusion of the intervention group. This design has the advantage that it reduces the risk of contamination and crossover between the two groups. Moreover, a crossover design is not possible given the progressive nature of AD. Furthermore, cluster randomization of hospitals is not feasible because of the differences in CAU between the sites. Since patients of both waves will be enrolled in the same sites, we assume that the waves will not show meaningful differences in demographic and clinical characteristics. Also, no substantial changes are expected in the upcoming years regarding current CAU in the memory clinics based on the view of collaborating experts and the organization of care in the last years.

Subjects will be followed for 6 months while undergoing three assessments during this period. The primary outcome will be the QoL of patients and their caregivers. Secondary outcomes include changes in the prevalence and severity of NPS, caregiver burden, caregivers’ confidence managing NPS, psychotropic medication use, the experiences of patients and caregivers who underwent the DICE method, and the cost-effectiveness of the intervention.

### Eligibility criteria

In order to be eligible to participate in this study, patients must meet all of the following criteria (see Table 1): (a) a clinical diagnosis of probable AD (NIA-AA criteria by McKhann et al. [41]) or MCI due to AD (NIA-AA criteria by Albert et al. [42]) with at least intermediate probability of AD etiology based on: patient history, neuropsychological assessment [43], and neuroimaging (magnetic resonance imaging (MRI) or positron-emission tomography (PET)). The clinical diagnosis needs to be established within the last 2 years so that patients with a diagnosis who visit the memory clinic for clinical follow-up might also participate; (b) presence of NPS established with the Neuropsychiatric Inventory Questionnaire (NPI-Q, presence of ≥ 1 symptoms) administered within the last month [44]; (c) a Mini-Mental State Examination (MMSE) score > 15 so that patients are able to reflect on their QoL [45]; (d) patients need to be community-dwelling; and (e) availability of a reliable informant who is considered to be the primary caregiver.

**Table 1:** Eligibility criteria

Inclusion criteria	Exclusion criteria
Diagnosis of MCI due to AD or AD dementia based on patient history, neuropsychological assessment, and neuroimaging within the last 2 years	Meeting the additional criteria of a non-AD neurodegenerative disease (vascular co-pathology is permitted)
Presence of NPS; ≥ 1 symptoms on NPI-Q	Legally incapable to give informed consent
MMSE score > 15	Evidence of current delirium or previous delirium
Patients need to be community-dwelling	Primary (premorbid) psychiatric disorders that could better explain the manifestation of NPS
Availability of a reliable informant	Participating in a clinical (medication) trial

*MCI* mild cognitive impairment, *AD* Alzheimer’s disease, *NPS* neuropsychiatric symptoms, *NPI-Q* Neuropsychiatric Inventory Questionnaire, *MMSE* Mini-Mental State Examination

A potential subject who meets any of the following criteria will be excluded from participation in this study: (a) patients meet the (additional) criteria of any non-AD neurodegenerative disease, except vascular co-pathology; (b) legally incapable (as judged by the attending physician and therefore unable to give a written consent; (c) evidence of current delirium or previous delirium in the past 6 months; (d) primary (premorbid) psychiatric disorders such as schizophrenia or bipolar disorder that could better explain the manifestation of NPS, or current abuse of alcohol or drugs; and (e) currently participating in a clinical trial. Patients are allowed to be on medication (e.g., acetylcholinesterase inhibitors or psychotropic drugs) prior to inclusion since no differences between the two waves are expected regarding the medication use at baseline, and this will be carefully documented in a case report form (CRF).

### Recruitment

Patients will be recruited from six different memory clinics in and around Rotterdam in the Netherlands (Erasmus University Medical Center, Franciscus Gasthuis & Vlietland, Het Van Weel-Bethesda Ziekenhuis, Maasstad Hospital, and Spijkenisse Medical Center) to facilitate patient enrollment and guarantee a good mixture of patients from both academic and general hospitals.

After a diagnosis of MCI or AD dementia is established at one of the memory clinics, study eligibility will be evaluated based on the in- and exclusion criteria by the local attending physician. Alternatively, patients already diagnosed with MCI or AD dementia who visit the memory clinic for clinical follow-up will also be identified based on these criteria.

### Interventions

#### Control group

Participants in the control group will receive CAU at their local hospital. We expect that the CAU will be quite heterogeneous over sites and may consist of psychoeducation about dementia by a nurse or consultant specialized in dementia, the prescription of psychotropic drugs, and/or the referral to a psychiatric outpatient clinic for specialized treatment in patients with severe NPS [46]. Because of these differences, we will carefully keep track of the procedures undertaken by clinicians for patients in the CAU group. Based on recommendations for assessing usual care in clinical trial [47], we will develop a study-specific CRF that will be filled out at the time of enrolment and will be updated at each follow-up visit.

#### Intervention group

All participants in the second wave will be enrolled in the intervention group. In this group, we will apply the DICE method to structure and standardize the assessment and management of NPS. Participants who withdraw from study participation after being informed by their physician and/or the researchers will receive CAU at their hospital as described above. The DICE method will take place at the Neurology Department of the Erasmus MC and will be carried out by a psychiatrist (MC) and neuropsychologists (EB, WSE, JMP) who are all involved in the memory clinic of this department.

**Fig. 2:**
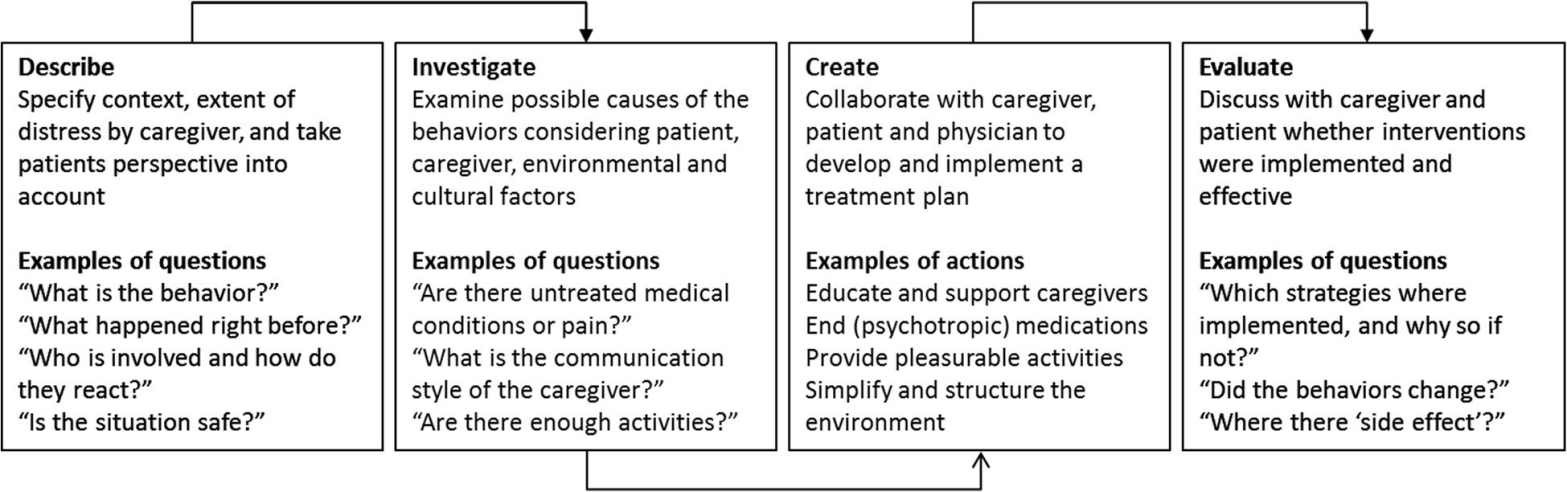
The DICE method with examples of questions and actions. Adapted from Kales et al. [10] and Gitlin et al. [48]

The steps of the DICE method are depicted in Fig. 2. More detailed information on the development and background of the DICE method can be found elsewhere [10]. During the first visit, the patient and caregiver will undergo a consultation by an experienced psychiatrist to establish clinically relevant NPS (*Describe*). Factors related to the patient, caregiver, and environment will be examined following the DICE method [22, 48] and the DICE manual [49]. For factors related to the patient, we will record the chronic somatic conditions using the Cumulative Illness Rating Scale for Geriatrics (CIRS-G) [50] semi-structured interview, followed by a clinical examination to explore the medication changes, pain, sleep hygiene, and sensory changes. If necessary, a lab evaluation will be conducted to screen for infections, thyroid problems, and metabolic disorders. Other patient-related factors including unmet needs, boredom, and emotional well-being will be assessed using the Checklist of Factors to Consider to Identify Potential Causes of Behavioral Symptoms developed by Gitlin et al. [48]. Caregiver-related factors will be screened by using the Relationship Closeness Scale [51], Center for Epidemiologic Studies Depression Scale [52], and the CareQol-7D [53]. This will be extended by history taking on family and cultural expectations, knowledge about dementia, and the availability of support. Environmental factors will be assessed to the patient and caregiver by the Informal Assessment: Brief Questions to Guide Describing Behavioral Symptoms [48]. A full and accurate description of specific behavior will provide more insight about the “who, when, where, and what” about the situations in which the behavior is occurring, while taking safety risks and the level of physical and social stimulation into account (*Investigate*). Thereafter, a multidisciplinary meeting will take place in which a personalized treatment advice is formulated based on the current guidelines on the diagnosis and assessment of NPS in dementia (*Create*) [24–26]. During the second visit, this treatment advice is discussed and adjusted to the needs, values, and characteristics of the patient and caregiver following a PCC approach. Given the large heterogeneity in symptoms, interventions will vary for each individual and can include psychoeducation, psychosocial interventions, caregiver support, and/or pharmacological treatment based on the current (inter)national guidelines [24–26, 54–56]. Notably, the interventions and strategies that will be used to reduce NPS and enhance the QoL are all evidence-based treatment strategies that are or should be carried out in the current clinical practice. Finally, we will monitor treatment progression 1 month after the last visit by telephone (*Evaluate*). Patients and their caregivers are then invited for an extra visit if necessary. In such cases, alternative interventions will be discussed if planned interventions were not implemented or effective. Additional diagnostic procedures or interventions will be monitored in the CRF.

### Outcome measures

For the control group, measurements will take place at baseline (T0), with follow-up testing at 3 months (T1) and 6 months (T2). For the intervention group, measurements will take place at baseline (T0), directly after treatment (T1), and follow-up at 6 months (T2). The T1 measurements will be planned after finishing the (psychosocial) intervention and/or when medication is stabilized and thus may vary between subjects in the intervention group. We will gather all relevant clinical and intervention-related information which enables us to study post hoc whether this variation in T1 assessments may have resulted in bias. All measurements will take place at the local hospitals or at the patients’ homes, see Table 2 for an overview of all outcome measures.

**Table 2:** Outcome measures

Outcome	Measure	Purpose	Respondent	Time of assessment
Demographic characteristics	For example, age, education, sex, ethnicity, relation to caregiver	Descriptive, covariate, moderator	Patient and caregiver	T0
Clinical characteristics	For example, diagnosis, AD biomarkers, disease duration	Descriptive, moderator	Patient and caregiver	T0*
Disease severity	CDR [57]	Descriptive, moderator	Patient	T0*, T2
Cognitive status	MMSE [58]	Descriptive, moderator	Patient	T0*, T2
Cognitive functioning	Standardized cognitive test battery [43]: RAVLT [59], VAT [60], DS [61], VF [62], LDST [63], SCWT [64], TMT [65]	Descriptive, moderator	Patient	T0*
Functional abilities	A-IADL-Q [66]	Descriptive, moderator	Caregiver	T0, T2
Psychotropic medications	Brown bag review [20]	Descriptive, secondary outcome	Caregiver	T0, T1, T2
Comorbidities	CIRS-G [50]	Descriptive	Patient	T0, T2
Quality of life patient	QoL-AD [67]	Primary outcome	Patient and caregiver	T0, T1, T2
Quality of life caregiver	CarerQol-7D [53]	Primary outcome	Caregiver	T0, T1, T2
Caregiver burden	Perseverance time [68]	Secondary outcome	Caregiver	T0, T1, T2
Caregiver competence managing NPS	Additional NPI-Q item [69]	Secondary outcome	Caregiver	T0, T1, T2
Cost-effectiveness	EQ-5D-5 L [70], ICECAP-O [71] iMTA iVICQ [72], iMTA MCQ [73]	Secondary outcome	Patient Caregiver	T0, T1, T2
NPS prevalence, severity, and distress	NPI-Q [44]	Secondary outcome	Caregiver	T0*, T1, T2
If NPI-Q frequency score ≥ 1 on:				
“Agitation,” “motor disturbances,” “irritability,” “disinhibition”	Cohen-Mansfield Agitation Inventory [74]	Secondary outcome	Caregiver	T0, T1, T2
“Apathy”	Apathy Evaluation Scale-I [75]	Secondary outcome	Caregiver	T0, T1, T2
“Depression,” “anxiety,” “elation”	CSDD [76], RAID [77]	Secondary outcome	Caregiver	T0, T1, T2
“Hallucinations,” “delusions”	BEHAVE-AD subscales psychosis, delusions [78]	Secondary outcome	Caregiver	T0, T1, T2
“Nighttime behaviors”	Sleep Disorder Inventory [79]	Secondary outcome	Caregiver	T0, T1, T2

*AD* Alzheimer’s disease, *CDR* Clinical Dementia Rating, *RAVLT* Rey Auditory Verbal Learning Test, *VAT* Visual Association Test, *DS* Digit Span, *VF* Verbal Fluency (animals), *LDST* Letter Digit Substitution Test, *SCWT* Stroop Color Word Test, *TMT* Trail Making Test, *MMSE* Mini-Mental State Examination, *A-IADL-Q* Amsterdam Instrumental Activity of Daily Living Questionnaire, *CIRS-G* Cumulative Illness Rating Scale for Geriatrics, *QoL-AD* Quality of Life in Alzheimer’s Disease, *iMTA iVIQ* iMTA Valuation of Informal Care Questionnaire, *iMTA MCQ* iMTA Medical Costs Questionnaire, *NPI-Q* Neuropsychiatric Inventory Questionnaire, *CSDD* Cornell Scale for Depression in Dementia, *RAID* Rating Anxiety In Dementia, *BEHAVE-AD* Behavioral Pathology in Alzheimer’s Disease Rating Scale

*Will be carried out during the diagnostic procedure at local hospitals

#### Primary outcomes

The QoL of the patient will be measured by the Quality of Life in Alzheimer’s Disease (QoL-AD) questionnaire [67]. This is one of the most widely used QoL questionnaires in AD and has good psychometric properties [80]. Patients are questioned via a 13-item interview format. The proxy version of the QoL-AD is also used and filled out by the caregiver since previous studies have shown that the caregivers’ perspective on the patients’ QoL might be a more valid indicator of treatment success [81].

The CarerQol-7D will be used to measure the care-related QoL in caregivers [53]. The instrument includes six burden dimensions and a subjective valuation scale for happiness.

#### Secondary outcomes

Changes in NPS will be assessed with the NPI-Q [44], a general screening questionnaire including 12 distinct NPS. For each item, caregivers have to indicate the presence, the severity, and the extent of emotional distress that each symptom causes. Similar to Gitlin et al. [69], we will add a frequency score and will ask caregivers how confident they are in managing a certain symptom on a 5-point Likert scale (0 = not confident to 4 = extremely confident).

A two-step approach will be used to further assess NPS: if certain symptoms are present, as indicated by an NPI-Q frequency score ≥ 1, specific questionnaires will be used to assess these symptoms in more detail. All instruments will be administered to the caregiver. To measure the depressive symptoms, the Dutch version of the Cornell Scale for Depression in Dementia (CSDD) will be used [76]. The CSSD consists of 19 items covering mood, behavioral changes, and circadian changes related to depression and is validated in patients with dementia [76]. Anxiety symptoms will be measured by the Rating Anxiety in Dementia (RAID) scale, an 18-item inventory that includes specific fears and somatic symptoms related to anxiety [77]. Agitation, irritability, aggression, and motor disturbances will be measured by the Dutch version of the Cohen-Mansfield Agitation Inventory (CMAI-D) [82]. Hallucinations will be assessed by the subscale B of the Behavioral Pathology in Alzheimer’s Disease Rating Scale (BEHAVE-AD) [78], and delusions will be assessed by the subscale A of the BEHAVE-AD. Apathy is assessed with the informant-reported Apathy Evaluation Scale (AES-I) [75] and comprises of 18 items. Sleep disturbances will be measured by the 8-item Sleep Disorder Inventory (SDI) [79], an expanded version of the sleep disturbances item of the NPI. Similar to the NPI, caregivers have to score each symptom of the SDI on frequency, severity, and caregiver distress.

Caregiver burden will be measured with the perseverance time, a one-item questionnaire that assesses caregiver burden by asking the period of time (in months) that the informal caregiver thinks he or she is able to maintain the care if the current situation remains stable [68]. This questionnaire is a good predictor for institutionalization [83].

The Clinical Dementia Rating Scale (CDR), MMSE, and a neuropsychological assessment will be administered during the diagnostic procedure at the local memory clinic prior to inclusion. The CDR includes six domains covering cognitive function and IADL associated with dementia [57]. Disease severity will be determined based on clinical diagnosis and CDR global score with MCI due to AD (CDR score 0.5), mild AD dementia (CDR score 1), and moderate to severe AD dementia (CDR score 2–3). Global cognitive function will be measured with the MMSE [58]. The neuropsychological assessment will be carried out according to the Dutch Parelsnoer Institute for Neurodegenerative Diseases [40] and covers the major cognitive domains including memory, attention, processing speed, language, visuospatial abilities, and executive functioning.

The Amsterdam Instrumental Activities of Daily Living Questionnaire (A-IADL-Q) is a proxy measure to detect problems in IADL in patients with dementia [66]. This tool is a reliable and valid instrument to detect changes in IADL over time.

Physical health and comorbidities of the patient will be assessed using the CIRS-G [50]. The severity of 14 common medical problems in the geriatric population (e.g., heart, liver, vascular diseases) will be judged by one of the researchers during a short interview with the patient and caregiver.

Psychotropic medication use will be documented in the CRF. The total number of medications used will be registered and classified according to the ATC coding: antidepressants, antipsychotics, hypnotics and sedatives, anxiolytics, and anti-dementia medications [20].

#### Cost-effectiveness

For the cost-effectiveness evaluation, patients will complete the EQ-5D-5 L, the most commonly used health-related QoL instrument [70], and the ICEpop CAPability measure for Older people (ICECAP-O), a 5-item well-being scale [71], and caregivers will fill out the CarerQol-7D. In addition, the Institute for Medical Technology Assessment Valuation of Informal Care Questionnaire (iMTA iVICQ) [72] will be used to assesses the amount, costs, and appraisal of the care provided by the caregivers. The iMTA Medical Consumption Questionnaire (iMTA MCQ) consists of 31 questions regarding healthcare utilization [73] and incorporates direct healthcare use of the patient. Both the iMTA MCQ and the iMTA iVICQ will be sent to the caregivers and can be completed at home.

#### Qualitative endpoint data

A random selection of one out of four of the dyads in the intervention group will be invited to participate in the qualitative part of this study, accounting for the site and disease stage. Semi-structured interviews will be conducted in order to achieve more insight into the experiences of participants who underwent the DICE method and what they considered as helpful elements. The interviews will be conducted face-to-face, will be audio-taped, and will last approximately 60 min. Interviews will be performed until saturation is reached, i.e., until no new concepts and themes are obtained [84], which we estimate to reach after we interviewed 15–20 patients with their caregivers [8, 85, 86]. Questions will be asked in an open non-directive manner, focusing on the subjects’ thoughts, feelings, and experiences. Topics include the subjects’ experience of the intervention, and which elements were considered to be effective and which not, with the aim to examine the efficacy of and experiences with the DICE method from the perspective of patients and caregivers.

### Sample size

In order to reach sufficient power to detect reliable and clinically relevant changes, we performed a power calculation using G*Power. The power calculation is based on the results of a recent meta-analysis by Kim and Park [17], on the effectiveness of PCC in a mix of institutionalized and community-dwelling patients, and the results of a pilot study on the effectiveness of the DICE method in community-dwelling patients and caregivers [36]. Both studies showed a moderate effect size for the effects of PCC interventions on QoL in patients with AD and their caregivers when compared to CAU. Since there are limited validated sample size calculation methods for the mixed model approach we aim to use [87], our calculation is based on a repeated measures ANOVA. Using G*Power, the required sample sizes were *N* = 86 and *N* = 46 for between- and within-group analyses, respectively, based on a power of 0.80 and an alpha of 0.05. After enquiry, the 6 recruitment centers suggested that at least 25 patients receive a MCI or AD diagnosis annually at each site. Though there are a high number of patients available at all 6 study sites, the participation of eligible dyads is expected to be 150 since not all patients will fulfill our criteria or will be willing to participate (based on an estimated inclusion rate of 40% [88]). We will carefully keep track of the reasons why eligible subjects refuse to participate. We will recruit a total of 150 patients during the total inclusion period of approximately 3 years (*N* = 75 in the control group and *N* = 75 in the intervention group), which exceeds the estimated needed sample size, even when accounting for dropout/loss to follow-up.

### Statistical methods

*T*-tests or chi-square tests will be used to identify the differences in baseline characteristics (e.g., age, education level, disease severity) between the control group and the intervention group. For the primary and secondary study outcomes, we will use an intention-to-treat approach including all subjects irrespective of the adherence to our intervention [89]. Thereafter, we will perform per-protocol analyses with only the subjects who completed the intervention (underwent all DICE steps). We will correct for multiple testing.

We will use the Little’s Missing Completely at Random Test to examine whether the data are missing at random or missing completely at random. The mixed model analyses will be able to handle the data when the missing data is completely at random. Multiple imputation will be used in cases when data is missing at random.

Linear mixed models will be used for the primary and secondary outcomes for the T0, T1, and T2 time points. These statistics are preferred when using longitudinal data because of its advantage in handling missing data and its capacity to deal with nested data and variance in follow-up duration between and within the groups. Changes in the trajectories of the primary and secondary outcomes are compared between the two groups. Subject, hospital, and time are considered as random effects, and baseline measure, group, and disease severity are accounted as fixed effects.

We will perform a cost-utility analysis of the intervention group versus the control group in accordance with the Dutch guidelines for economic evaluations on the basis of questionnaires [90].

Quality and length of life will be combined into quality-adjusted life years (QALYs) using a Markov model to extrapolate lifetime outcomes based on the data from this study combined with literature data. The EQ-5D-5 L, ICECAP-O, and CarerQol-7D data will be transformed into QALYs for patients and caregivers (well-being years for ICECAP-O), using published tariffs obtained from general reference populations [90, 91].

With the simple Markov model, we will calculate the incremental effectiveness of the DICE method versus the control group in QALYs, incremental costs, and the incremental cost-effectiveness ratios. We will also perform one-way, two-way, and probabilistic sensitivity analyses to determine the effect of uncertainty in all input parameters. Using a non-parametric bootstrapping (randomly drawing 5000 observations with replacement from the patient sample), the degree of uncertainty for costs and health effects and the cost-utility ratio will be depicted. In addition, an acceptability curve will be drawn, which indicates the probability that the intervention studied has lower incremental costs per QALY gained than various thresholds.

A budget impact analysis will be performed that includes relevant features and tariffs of the Dutch healthcare system; anticipated uptake of the new intervention as well as usual care will be considered. The budget impact per year of implementing the new intervention will be estimated. All elements of medical costs for the intervention group and the control group will be considered and calculated.

#### Qualitative analyses

The audiotapes of all interviews will be transcribed verbatim. This data will be analyzed by two independent researchers with ATLAS.ti 7 software according to the thick analysis approach [92]. This approach endorses multiple triangulations, i.e., the use of multiple interpreters and techniques to analyze the data, to enhance validity.

The coding and analyses will be an iterative process simultaneously with the interviews, allowing adjustment of questions and topics. We will make use of open coding, thematic coding, and causal coding [93]. Open coding is an explorative process in which all elements of the data are coded. Thematic coding is a more deductive technique that included the coding of themes and categories that are proposed by the researchers prior to the analysis or emerge from the material and are considered to be of importance by the researchers. Causal coding will help us to get more insight into the working elements of the DICE approach as proposed by the participants. Characteristics of patients and their caregivers (age, sex, relationship, disease severity) will be used for descriptive purposes.

## Discussion

The current paper describes the protocol of the BEAT-IT study, a multicenter study designed to investigate the effectiveness of a comprehensive assessment and personalized treatment of NPS in AD, following the DICE method to improve the QoL in patients with MCI and AD in the memory clinic. We hypothesize that early recognition and tailored treatment of NPS will benefit the QoL of patients and their caregivers; will reduce NPS, caregiver burden, and psychotropic drug use; and will lead to cost-effective care.

The novelty of this study lies in the inclusion of the whole spectrum of NPS, the enrollment of both patients with MCI and AD, and the evaluation of an approach that integrates both nonpharmacological and pharmacological interventions in the memory clinic setting. Besides standardized quantitative measures, a qualitative approach will be used to examine its efficacy and feasibility from the perspective of caregivers and patients. Also, important additional information will be obtained from studying the first wave of participants, enabling us to examine “naturalistic” progression of NPS and its relationship with other clinical measures. Insight in the current CAU of NPS will aid us in the formulation of recommendations to improve the daily clinical practice regarding the care of NPS in AD. After establishing the effectivity of the DICE method in the memory clinic setting, a next step would be to examine the implementation of this approach at other sites by taking already suggested and unique local barriers into account.

At the time of writing, recruitment is ongoing and is expected to be completed in December 2019 for the control group. Hereafter, the intervention group will be enrolled until the beginning of 2021, and follow-up measures will be completed in autumn 2021. Results will be available in late 2021.

There are a few possible threats to this study. Firstly, the use of the NPI-Q to screen for eligible patients might introduce an observer bias since this measure is not part of the regular diagnostic workup at some sites. Consequently, NPS may be detected more often, resulting in care that may not fully reflect the current CAU, i.e., underestimating the expected underrecognition and undertreatment of NPS in AD. Second, the current guidelines consider psychosocial interventions as the first-line treatment but mainly suggest interventions that may be more suitable for institutionalized patients with severe dementia, e.g., reminiscence therapy, aromatherapy, or “snoezelen” [24, 25]. Although various nonpharmacological interventions have been shown to be effective in community-dwelling patients [18], these strategies are rarely mentioned in the guidelines and therefore not integrated in clinical practice [94]. For our interventions, we will select nonpharmacological strategies based on prior studies (e.g., [48]) and our clinical expertise. Third, our outcome measures are mainly based on self-reported questionnaires that may not fully capture all effective aspects of the intervention [95, 96]. Moreover, patients with dementia may have difficulties completing the QoL questionnaires (EQ-5D-5 L, ICECAP-O) due to cognitive problems [97]. To circumvent some of these problems, we will also use qualitative research methods which enables us to better understand and measure the QoL of patients and to give participants the opportunity to express their experiences with the DICE method in an unrestricted manner. Fourth, the substantial differences across sites in CAU might be a challenge to this study, as patients visiting certain sites may receive more and different treatments compared to other centers. We will therefore aim to record all valuable information through our CRF, which enables us to perform post hoc sensitivity analyses, and verify whether this heterogeneity might affect the results. A final issue might be that patients are included based on clinical diagnostic criteria, without the use of AD pathophysiological biomarkers (e.g., abnormal levels of Aβ or tau proteins in CSF or on PET). Despite the fact that an MRI or (FDG-)PET scan of the brain is required, this may lead to the inclusion of patients who do not have underlying AD pathology, especially in those with MCI. However, the applied diagnostic criteria resemble those that are used in clinical practice where AD pathophysiological biomarkers are not part of the standard diagnostic workup. In addition, since this is a clinical study targeting clinical symptoms rather than the underlying disease process, we argue that the effects might be similar in patients with other underlying etiologies. We will however perform a sensitivity analysis in a subgroup of patients with positive AD biomarkers in order to study whether the effects are similar in this subgroup compared to the whole study group.

To conclude, the BEAT-IT study as a whole will increase our knowledge of the underlying neurobiology of NPS in AD, which may enable us to identify potential targets for therapeutic agents. The intervention study might provide evidence on how to structure and standardize the care of NPS in AD to improve the QoL of both caregivers and patients. Moreover, the findings of the intervention study will result in recommendations to improve the early detection and treatment of NPS in AD in the memory clinic.

## Abbreviations

AD: Alzheimer’s disease
AES-I: Apathy Evaluation Scale Information version
A-IADL-Q: Amsterdam Instrumental Activities of Daily Living Questionnaire
BEHAVE-AD: Behavioral Pathology in Alzheimer’s Disease Rating Scale
bvAD: Behavioral variant of AD
CAU: Care as usual
CDR: Clinical Dementia Rating Scale
CIRS-G: Cumulative Illness Rating Scale for Geriatrics
CMAI-D: Dutch version of Cohen-Mansfield Agitation Inventory
CRF: Care report form
CSDD: Cornell Scale for Depression in Dementia
CSF: Cerebrospinal fluid
FDG-PET: Fluorodeoxyglucose positron-emission tomography
IADL: Instrumental activity of daily living
ICECAP-O: ICEpop CAPability measure for Older people
iMTA iVICQ: Institute for Medical Technology Assessment Valuation of Informal Care Questionnaire
iMTA MCQ: Institute for Medical Technology Assessment Medical Consumption Questionnaire
MCI: Mild cognitive impairment
MMSE: Mini-Mental State Examination
MRI: Magnetic resonance imaging
NPI-Q: Neuropsychiatric Inventory Questionnaire
NPS: Neuropsychiatric symptoms
PCC: Patient-centered care
PET: Positron-emission tomography
QALY: Quality-adjusted life years
QoL: Quality of life
QoL-AD: Quality of Life in Alzheimer’s Disease questionnaire
RAID: Rating Anxiety in Dementia
SDI: Sleep Disorder Inventory
